# Transcriptomic and Functional Studies of the RGS Protein Rax1 in *Aspergillus fumigatus*

**DOI:** 10.3390/pathogens9010036

**Published:** 2019-12-31

**Authors:** Yong-Ho Choi, Min-Woo Lee, Olumuyiwa Ayokunle Igbalajobi, Jae-Hyuk Yu, Kwang-Soo Shin

**Affiliations:** 1 Department of Microbiology, Graduate School, Daejeon University, Daejeon 34520, Korea; youngho1107@gmail.com; 2 Soonchunhyang Institute of Medi-bio Science, Soonchunhyang University, Chungcheongnam-do 31151, Korea; mwlee12@sch.ac.kr; 3 Department of Microbiology, Institute for Applied Biosciences, Karlsruhe Institute of Technology, Fritz-Haber Weg 4, D-76131 Karlsruhe, Germany; olumuyiwa.igbalajobi@kit.edu; 4 Departments of Bacteriology and Genetics, University of Wisconsin-Madison, Madison, WI 53706, USA; 5 Department of Systems Biotechnology, Konkuk University, Seoul 05029, Korea

**Keywords:** *Aspergillus fumigatus*, GliM, metacaspase, Rax1, RNA-seq

## Abstract

In the comparative transcriptomic studies of wild type (WT) and *rax1* null mutant strains, we obtained an average of 22,222,727 reads of 101 bp per sample and found that 183 genes showed greater than 2.0-fold differential expression, where 92 and 91 genes were up-and down-regulated in Δ*rax1* compared to WT, respectively. In accordance with the significantly reduced levels of *gliM* and *casB* transcripts in the absence of *rax1*, the Δ*rax1* mutant exhibited increased sensitivity to exogenous gliotoxin (GT) without affecting levels of GT production. Moreover, Δ*rax1* resulted in significantly restricted colony growth and reduced viability under endoplasmic reticulum stress condition. In summary, Rax1 positively affects expression of *gliM* and metacaspase genes.

## 1. Introduction

The G protein signaling pathway plays central roles in transmitting environmental stimuli into intrinsic signals leading to appropriate gene expression changes. Binding of an external ligand to an associated G protein coupled receptor (GPCR) causes GDP-to-GTP exchange of a Gα, which then results in dissociation of Gα-GTP and Gβγ subunits. Dissociated (activated) G protein subunits interact with downstream effectors leading to activation of various signaling pathways involving cAMP-dependent protein kinase A (PKA), mitogen activated protein kinases (MAPKs), or protein kinase C (PKC) [1,2,3]. One of the key control points of these signaling pathways is at the G protein level exerted by regulators of G protein signaling (RGSs). Normally, an RGS protein interacts with its target Gα-GTP subunit and activates intrinsic GTPase activity of the Gα-GTP, leading to accelerated GTP to GDP hydrolysis. Gα-GDP then associates with its Gβγ subunit, leading to turn-off of the G protein-mediated signaling pathway [2,4].

In the genome of the opportunistic human pathogenic fungus *Aspergillus fumigatus*, six RGS proteins that contain the conserved RGS domain have been identified based on homology searches, and the roles of them have been characterized [5,6,7,8,9,10]. Previously, we reported that Rax1 of *A*. *fumigatus* positively controls vegetative growth and asexual development. In addition, the *rax1* null mutant conidia were more resistant against exogenous H_2_O_2_ via elevated accumulation of intracellular trehalose and cell wall melanin [7]. In the present study, to further understand the functions of Rax1, we carried out RNA-Seq-based transcriptome analyses of wild types (WT) and *rax1* null mutants. In addition, follow-up functional studies corroborating the findings of genome-wide expression analyses are reported.

## 2. Results

### 2.1. Summary of the RNA-Seq Data

**Figure 1:**
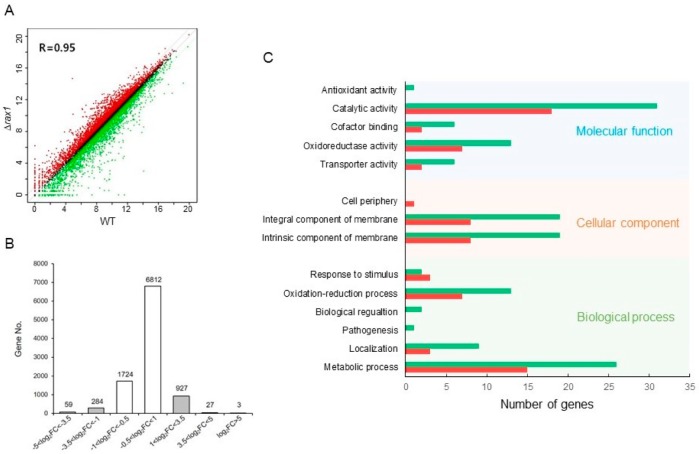
Summary of the transcriptomic analyses of wild type (WT) and Δ*rax1* strains. (**A**) Linear fitted model showing the correlation of overall gene expression between WT and Δ*rax1* strains. The correlation coefficient (R = 0.95) is indicated. (**B**) Histograms showing general transcriptomic results where the white bars fall in the −1 < log_2_FC < 1 fragment count range with low differential expression values. (**C**) Functional categories of DEGs. Genes associated with increased mRNA levels in the Δ*rax1* strain are represented with red bars and genes associated with decreased mRNA levels in the mutant strain are represented with green bars.

For the comparative RNA-seq analysis between WT and Δ*rax1* strain, cells were collected at 12 h cultures as described in Materials and Methods. Correlation of overall gene expression between WT and Δ*rax1* strain was highly correlated (R = 0.95, Figure 1A). The *A*. *fumigatus* genome contains 9840 protein, 205 tRNA, 34 rRNA, 29 snoRNA, 12 ncRNA, and 9 snRNA coding genes with 1 pseudogene (AspGD: http://www.aspergillusgenome.org). We obtained an average of 22,222,727 reads and 9,836 different transcripts for Δ*rax1* strain. Of the 9,836 genes, 1,300 genes (13.2%) displayed more than 2.0-fold (log_2_FC = 1.0) changes in mRNA levels (Figure 1B), of which 957 (9.7%) exhibited higher transcript levels in the Δ*rax1* strain than in the WT strain whereas 343 (3.5%) were down-regulated in the Δ*rax1* strain. Among them, statistically significant (*p* < 0.05) genes were 92 and 91, respectively (Tables S1 and S2).

### 2.2. Functional Category Analysis

Functional category analysis was performed by determining gene ontology (GO) terms that were enriched in differentially expressed genes (DEGs). The most represented molecular function GO categories are “Catalytic activity”, with 31 down- and 18 up-regulated, and “Oxidoreductase activity” with 13 up- and 7 down-regulated. The top significant cellular component GO categories are “Intrinsic and integral component of membrane” (19 up, 8 down). The most significant biological process GO categories are “Metabolic process” (26 up, 15 down), “Oxidation-reduction process” (13 up, 7 down), “Localization” (9 up, 3 down), and “Response to stimulus” (2 up, 3 down) (Figure 1C). Table S1 lists the at least 2.0-fold (log_2_FC = 1.0) up-regulated genes with (*p* < 0.05) following the deletion of *rax1*. The highest up-regulated gene was an emopamil binding protein (EBP) domain protein encoding gene (AFUA 1G15100), which is involved in sterol metabolic process and a majority of up-regulated genes encodes hypothetical proteins. Most of the down-regulated genes were transcription factors and transporters (Table S2), including Zn(II)2Cys6 transcription factors (AFUA 7G05080, 8G02280, 4G01322, and 2G04860) and major facilitator superfamily (MFS) transporters (AFUA 7G05190, 3G00540, 6G02400, 3G02060, 2G12500, 2G17360, 3G01670, and 3G03190).

### 2.3. Rax1 Functions in the Resistance against Exogenous Gliotoxin

**Figure 2:**
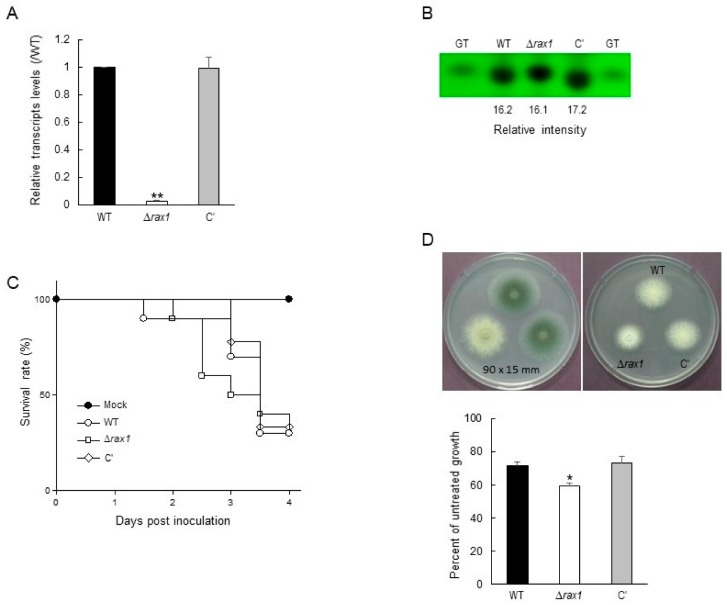
Roles of Rax1 in gliotoxin (GT) production, virulence, and tolerance to exogenous GT. (**A**) Levels of gliM transcript analyzed by qRT-PCR. (**B**) GT production in WT, Δ*rax1*, and C′ strains. The culture supernatant of each strain was extracted with chloroform and subjected to TLC. (**C**) Kaplan–Meier plots of the survival of ICR mice after infection with three strains of conidia. (**D**) Effect of exogenous GT on the growth of three strains of conidia. Conidia (1 × 10^5^) of each strain was inoculated in glucose minimal medium (MMG) containing GT (10 µg/mL) and incubated at 37 °C for 48 h. Statistical differences were evaluated with an ANOVA test. * *p* < 0.05.

The most down-regulated gene by Δ*rax1* was the one encoding the *O*-methyltransferase GliM (Log_2_FC = −10.83). To assess RNA-seq result, we estimated the mRNA levels of *gliM* by qRT-PCR. As shown in Figure 2A, level of *gliM* transcript in mutant strain was significantly lower (about 0.025-fold) than that of WT. GliM catalyzes the conversion of reduced dithiol gliotoxin (GT) to bisthiomethyl gliotoxin and plays a role in protecting fungi against dithiol containing toxins [11]. The production of GT analyzed by TLC and the relative intensity of GT of WT, Δ*rax1*, and complemented (C′) strains was 16.2, 16.1, and 17.2, respectively, implying that GT production was not affected by the absence of *rax1* (Figure 2B). We also checked the effect of Rax1 on the virulence in the CD1 (ICR) female mice. Conidia of three strains were inoculated in mice, and monitored every 12 h for survival for 4 days after challenge. The virulence of Δ*rax1* strains was avirulent compared to WT and C′ strains (Figure 2C). Comparison of survival curves analyzed by a log rank test revealed that there was no meaningful difference between the three strains (*p* = 0.7919). To test the effect of reduced expression of *gliM* in the Δ*rax1* strain, we checked the tolerance of the mutant to exogenous GT in comparison to WT. When conidia of three strains were inoculated in the presence of GT, the radial growth of all strains was decreased compared to untreated controls. While the growth of WT and C’ strains were decreased about 30%, which of Δ*rax1* strain was decreased about 40% (Figure 2D), indicating that the Δ*rax1* mutant was more sensitive than WT and C’ strains against exogenous GT. These results suggest that Rax1 is required for proper expression of *gliM* leading to the self-protection against exogenous GT, but not de novo biosynthesis of GT.

### 2.4. Rax1 Positively Regulates Metacaspase Activity

**Figure 3:**
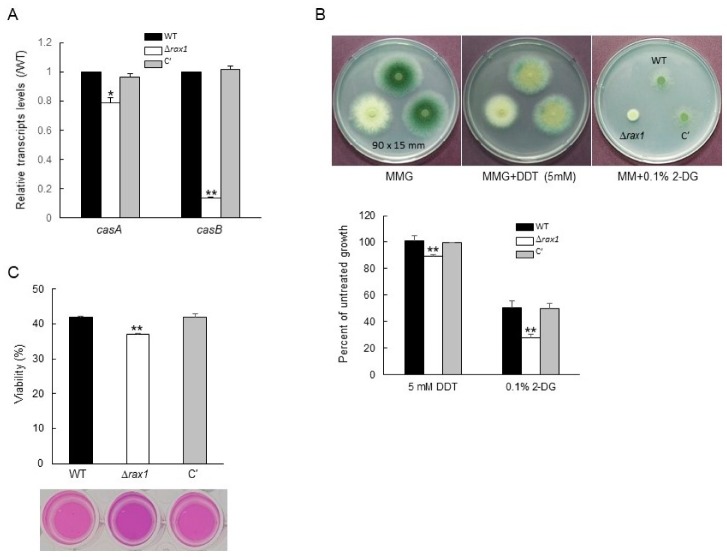
The Δ*rax1* mutant shows enhanced sensitivity to ER stressors. (**A**) qRT-PCR analysis of the metacaspase genes in WT, Δ*rax1*, and C′ strains. (**B**) Same numbers of conidia (5 × 10^5^) from the three strains were spotted into 5 mM dithiothreitol (DTT) and 0.1% 2-deoxy-D-glucose (2-DG) containing media and determined radial growth rate. (**C**) Approximate 10^4^ conidia were inoculated into 2 mL of glucose minimal medium (MMG) containing brefeldin A (BFA), incubated at 37 °C for 48 h, and detected cell viability with alamarBlue (AB). Statistical differences were evaluated with an ANOVA test. ** *p* < 0.01.

In RNA-seq analysis, we found that the transcript encoding the metacaspase CasB was significantly down-regulated (Log_2_FC = −2.90) in the Δ*rax1* mutant compared to WT (Table S3). To test whether Rax1 affects metacaspases expression, qRT-PCR analysis for the *casA* and *casB* transcript was carried out. The deletion of *rax1* led to reduced mRNA levels of metacaspase encoding genes (Figure 3A). To test sensitivity against endoplasmic reticulum (ER) homeostasis disrupting agents, radial growth and survival rates of three strains were determined in the presence of dithiothreitol (DTT), 2-deoxy-D-glucose (2-DG), and brefeldin A (BFA). The radial growth of the mutant was reduced by the treatment of DTT and 2-DG (Figure 3B). Markedly, the Δ*rax1* mutant exhibited significantly restricted vegetative growth on the medium containing 0.1% 2-DG as the sole carbon source (about 50% of WT and C′ strains) (Figure 3B). Against another ER stressor BFA, as shown in Figure 3C, while viability of WT and C′ strains was about 47%, but that of Δ*rax1* strain was 35% indicating that it is likely that Δ*rax1* might lead to reduced tolerance to BFA (Figure 3C).

## 3. Discussion

RGSs are a group of proteins including conserved RGS box that interacts with a GTP-Gɑ subunit and turn-off the G protein signaling pathways [4]. One of the *A*. *fumigatus* RGS, Rax1 (RgsB) protein, is similar to ScRax1P of the yeast *Saccharomyces cerevisiae*, where ScRax1P is required for the establishment of the bipolar budding [12]. However, the function of its homologous proteins is poorly understood in filamentous fungi. Previously, we reported that Rax1 of *A*. *fumigatus* positively controls growth, development, and oxidative stress response [7]. To further understand the functions of Rax1, we carried out RNA-Seq-based genome-wide expression studies and additional functional investigation.

Based on the transcriptome analysis, we found that a large number of genes are down-regulated in the Δ*rax1* strain compared to the WT strain. At first, we focused on the most down-regulated gene, *gliM*. The *gliM* gene is located in the GT biosynthetic gene cluster that comprises 13 genes [13,14] and encodes an *O*-methyltransferase. Disruption of *gliM* completely abrogated *bis*-methyl gliotoxin biosynthetic ability, while GT production and secretion was unaffected [11]. By modification of the active dithiol form of GT to an inactive *bis*-thiomethylated form, the GT producing fungi are protected from dithiol end products and/or toxic biosynthetic intermediates. In accordance with these, while there were no significant differences in the production of GT between strains, growth of the Δ*rax1* strain was more inhibited by exogenous GT compared to WT and C′ strains (Figure 2).

The caspase plays a central role in apoptosis and can be classified into the metazoan caspase family, paracaspase family, and metacaspase family [15]. The metacaspase family is specific to plant, fungi, and protozoa. *A*. *fumigatus* has two metacaspases, CasA and CasB, which function in both apoptotic morphology and resistance to ER stress [16]. The metacaspase deletion mutants showed a growth detriment in ER stress conditions [16]. In RNA-seq and qRT-PCR analyses, the expression of both metacaspases was significantly decreased by loss of *rax1* (Figure 3A, Table S2). Moreover, growth of the Δ*rax1* strain was impaired on medium containing DTT and 2-DG, which induces ER stress [17,18]. The sensitivity to ER stressor was confirmed using BFA, which generates ER stress by blocking the transport of proteins to the Golgi apparatus [19]. Viability of the Δ*rax1* strain was significantly reduced compared to WT and C′ strains in the presence of BFA (Figure 3C). Metacaspase-deficient mutants showed similar results. Loss of either *casA* or *casB* resulted in impaired radial growth on medium containing 2-DG and tunicamycin that induces ER stress in fungi [18,20,21], and loss of both genes had an additive effect. From these results, it can be concluded that Rax1 is required for normal growth of *A*. *fumigatus* in the ER homeostasis perturbation conditions by positively regulating CasA and CasB.

## 4. Materials and Methods

### 4.1. Strains and Culture Conditions

Glucose minimal medium (MMG) and MMG with 0.1% yeast extract (MMY) with appropriate supplements were used for culture of wild type (AF293) and the *rax1* null mutant strains [7]. Conidia were propagated MMY for 5 days at 37 °C and were collected with 0.02% Tween 80. The suspension was passed through a Miracloth (Millipore, Burlington, MA, USA) to remove hyphae and counted using a hemocytometer. To examine production of secondary metabolites, spores of relevant strains were inoculated at a final concentration of 5 × 10^5^ conidia/mL to 50 mL of liquid MMY with appropriate supplements and incubated at 250 rpm at 37 °C for 4 days.

### 4.2. Nucleic Acid Manipulation

Total RNA isolation and quantitative RT-PCR (qRT-PCR) assays were was carried out as previously described [5,22]. Briefly, conidia (5 × 10^5^ conidia/mL) of WT, Δ*rax1,* and C strains were inoculated into MMY liquid media with appropriate supplements and incubated at 37 °C, 250 rpm. Mycelial samples were collected and squeeze-dried at indicated time points. The sample was homogenized using a Mini Bead beater in the presence of silica/zirconium beads (0.3 mL, BioSpec Products, Bartlesville, OK, USA) and TRIzol^®^ reagent (1.0 mL, Invitrogen, Carlsbad, CA, USA). Quantitative RT-PCR (qRT-PCR) assays were carried out according to the manufacturer’s instruction using a Rotor-Gene Q (Qiagen, Hilden, Germany). Each run was assayed in triplicate with the RNA template, One Step RT-PCR SYBR Mix (MGmed, Seoul, Korea), reverse transcriptase (MGmed, Korea), and 10 pmole of each primer (Table S3). The data were normalized to *ef1α* expression [10] and calculated according to the ΔΔ*C*t method [22].

### 4.3. RNA-Seq Experiment

For RNA-seq analyses, cellophane sheet was spread out on the agar medium and 50 μL of a conidial suspension (10^7^/mL) was inoculated onto the cellophane. The plates were incubated in the dark at 37 °C for 12 h. We collected fungal colonies in triplicate and they were immediately frozen in liquid nitrogen and ground into a fine powder with a mortar and pestle. Total RNA was extracted as in the above described method. After analysis of RNA quality, three RNA samples from the same strain were combined and analyzed. This was done in duplicate. Total RNA was submitted to eBiogen Inc. (Seoul, Korea) for further analyses. RNA-Seq reads were mapped using TopHat software v2.1.0 tool in order to obtain the alignment file and *Aspergillus fumigatus* Database (NCBI) was used as the template for mapping. The alignment file was used to assemble transcripts, estimate their abundances, and detect differential expression of genes or isoforms using Cufflink v2.2.1. Gene classification was done based on searches done by BioCarta (http://www.biocarta.com/), GenMAPP (http://www.genmapp.org/), DAVID (http://david.abcc.ncifcrf.gov/), and Medline databases (http://www.ncbi.nlm.nih.gov/).

### 4.4. Phenotype Analyses

The tolerance to gliotoxin (GT, Sigma–Aldrich, Saint Louis, MO, USA) was tested with conidia of WT and mutant strains. Each conidia was inoculated onto MMG plates with the presence or absence of GT (10 µg/mL) and incubated at 37 °C for 48 h [14]. GT was extracted with chloroform as described previously [23] and analyzed by a thin-layer chromatography (TLC) silica plate (Kiesel gel 60, E. Merck). The TLC plate was developed with chloroform:methanol (9:1, *v*/*v*). For test of ER stress responses, DTT (5 mM) was added into MMG and 2-DG was used in place of glucose at 0.1% (*w*/*v*) in MMG solid medium. To assess sensitivity to BFA, conidia (1 × 10^4^) were inoculated into 2 mL of MMG containing BFA (1×) and incubated at 37 °C for 48 h. Cell viability was determine using alamarBlue (AB) assay based on the percent reduction of AB as described previously [24].

### 4.5. Murine Virulence Assay

For the persistently neutropenic mouse model, we used outbred ICR (Orient Bio Inc., Seongnam-si, Korea) female mice (30 g in body weight, 6 to 8 weeks old), which were housed five per cage and had access to food and water ad libitum. Mice were immunosuppressed with intraperitoneal injections (i.p.) of cyclophosphamide (Sigma–Aldrich, USA) at 250 mg/kg for 4 days prior to infection and with cyclophosphamide at 250 mg/kg and cortisone acetate (Sigma–Aldrich, USA) injected subcutaneously at 250 mg/kg 1 day prior to infection. At day 1 and 3 day post-infection, administrations were repeated with cyclophosphamide (125 mg/kg i.p.). Mice were anesthetized with isoflurane and then intranasally infected with 1 × 10^7^ conidia of *A*. *fumigatus* strains (10 mice per each strain) in 30 µL of 0.01% Tween 80 in PBS. Mice were observed every 12 h for survival for 4 days after challenge. Mock mice were inoculated with sterile 0.01% Tween 80 in PBS.

### 4.6. Statistical Analyses

Comparison of mRNA expressions, radial growth, and viability within the different strains were performed by one-way ANOVA tests. All results were expressed as means ± standard deviation (SD) and a *p* value less than 0.05 was considered statistically significant. Analyses of survival rates were performed by creating Kaplan–Meier plots and then performing log rank tests (GraphPad Prism 7.0). And comparisons for survival studies were considered significant if the *p* value was <0.05.

### 4.7. Ethics Statement

All of the animal procedures in this study were reviewed and approved by the Institutional Animal Care and Use Committee of Daejeon University (DJUARB2019-024).

### 4.8. Data Availability

The RNA-seq data are available from NCBI Gene Expression Omnibus (GEO) database under the accession number GSE100101.

## Supplementary Materials

The following are available online at https://www.mdpi.com/2076-0817/9/1/36/s1. Table S1: Up-regulated genes in Δ*rax1* relative to WT (*p* < 0.05), Table S2: Down-regulated genes in Δ*rax1* relative to WT (*p* < 0.05), Table S3: Oligonucleotides used in this study.
