# Supplementary material for: Transcriptomic and Functional Studies of the RGS Protein Rax1 in Aspergillus fumigatus
Source: Pathogens. 2019 Dec 31;9(1):36. doi: 10.3390/pathogens9010036 (PMC7168642; doi:10.3390/pathogens9010036)
Supplement: Supplementary file 1 [file pathogens-09-00036-s001.zip › New folder/Table S1.pdf]

Table S1. Up-regulated genes in Δ*rax1* relative to WT (> 2.0-fold, $p < 0.05$).

Gene name	Log_2FC	*p*-value	*q*-value	Protein product	Protein name
AFUA_1G15100	3.92	0.000	0.029	XP_752879.1	EBP domain protein
AFUA_3G03080	3.81	0.004	0.000	XP_748630.1	endo-1,3(4)-beta-glucanase
AFUA_1G14480	3.42	0.013	0.000	XP_752817.1	hypothetical protein AFUA_1G14480
AFUA_6G08500	2.89	0.002	0.046	XP_750739.2	phosphoglycerate mutase family protein
AFUA_5G03770	2.88	0.000	0.035	XP_747967.1	lipase
AFUA_4G06620	2.87	0.000	0.009	XP_752164.1	Glutamate/Leucine/Phenylalanine/Valine dehydrogenase
AFUA_2G17920	2.73	0.002	0.001	XP_756129.1	conserved hypothetical protein
AFUA_1G14190	2.70	0.005	0.012	XP_752789.1	DUF614 domain protein
AFUA_4G01270	2.68	0.000	0.030	XP_746331.1	integral membrane protein
AFUA_1G14540	2.68	0.002	0.000	XP_752823.1	oxidoreductase, short-chain dehydrogenase/reductase family
AFUA_1G15070	2.50	0.000	0.016	XP_752876.1	mtDNA inheritance protein Dml1
AFUA_1G15180	2.48	0.000	0.001	XP_752887.1	conserved hypothetical protein
AFUA_8G01110	2.45	0.010	0.034	XP_747095.1	integral membrane protein
AFUA_1G14640	2.39	0.004	0.023	XP_752833.1	conserved hypothetical protein
AFUA_1G14130	2.34	0.005	0.013	XP_752783.1	conserved hypothetical protein
AFUA_1G14600	2.27	0.010	0.007	XP_752829.1	transcription initiation factor TFIID, 31kD subunit
AFUA_3G06020	2.26	0.000	0.000	XP_755004.1	glyoxalase family protein
AFUA_3G00680	2.25	0.000	0.017	XP_748389.1	copper amine oxidase
AFUA_3G01360	2.08	0.000	0.000	XP_748457.1	siderochrome-iron transporter
AFUA_5G08180	2.05	0.000	0.000	XP_753818.1	cell wall protein
AFUA_1G13830	1.96	0.018	0.004	XP_752753.1	threonine-rich protein

AFUA_3G00620	1.96	0.046	0.000	XP_748382.1	zinc-containing alcohol dehydrogenase
AFUA_4G09600	1.90	0.000	0.003	XP_751866.1	GPI anchored protein
AFUA_4G13800	1.83	0.001	0.004	XP_751452.2	extracellular sialidase/neuraminidase
AFUA_8G01860	1.82	0.004	0.002	XP_747020.2	NmrA-like family protein
AFUA_8G01960	1.82	0.014	0.003	XP_747010.1	conserved hypothetical protein
AFUA_7G06440	1.80	0.003	0.023	XP_748866.1	F-box domain protein
AFUA_8G02250	1.79	0.000	0.000	XP_746981.1	conserved hypothetical protein
AFUA_5G02770	1.78	0.000	0.000	XP_748065.1	conserved hypothetical protein
AFUA_6G03870	1.77	0.000	0.000	XP_747690.2	tetratricopeptide repeat protein
AFUA_1G13980	1.77	0.003	0.000	XP_752768.1	conserved hypothetical protein
AFUA_1G15200	1.74	0.000	0.026	XP_752889.1	Hsp70 family protein
AFUA_1G14490	1.71	0.003	0.000	XP_752818.1	aminotransferase
AFUA_1G17290	1.66	0.000	0.000	XP_753097.1	conserved hypothetical protein
AFUA_3G12982	1.65	0.002	0.045	XP_001481626.1	extracellular serine rich protein
AFUA_4G06610	1.65	0.000	0.005	XP_752165.1	conserved hypothetical protein
AFUA_8G00160	1.64	0.000	0.000	XP_747188.1	conserved hypothetical protein
AFUA_6G07150	1.60	0.000	0.000	XP_750608.1	actin binding protein
AFUA_1G14770	1.59	0.015	0.004	XP_752846.1	tRNA dihydrouridine synthase
AFUA_2G17140	1.59	0.000	0.001	XP_756048.1	conserved hypothetical protein
AFUA_1G14440	1.57	0.000	0.009	XP_752814.1	cation diffusion facilitator 1
AFUA_1G16620	1.56	0.000	0.044	XP_753029.1	C6 transcription factor
AFUA_3G03960	1.53	0.002	0.027	XP_748716.1	conserved hypothetical protein
AFUA_1G17310	1.53	0.000	0.000	XP_753099.1	MFS lactose permease

AFUA_2G10480	1.53	0.001	0.002	XP_755381.2	conserved hypothetical protein
AFUA_5G06790	1.50	0.001	0.009	XP_753954.1	conserved hypothetical protein
AFUA_2G05635	1.49	0.002	0.000	XP_001481660.1	hypothetical protein AFUA_2G05635
AFUA_3G00280	1.48	0.001	0.015	XP_748350.1	metallo-beta-lactamase domain protein
AFUA_1G01000	1.48	0.021	0.046	XP_749850.1	oxidoreductase, 2OG-Fe(II) oxygenase family
AFUA_8G01540	1.46	0.012	0.014	XP_747052.1	hypothetical protein AFUA_8G01540
AFUA_2G03780	1.46	0.007	0.013	XP_749510.1	conserved hypothetical protein
AFUA_2G01610	1.45	0.001	0.000	XP_749296.1	conserved hypothetical protein
AFUA_8G00200	1.44	0.000	0.022	XP_747184.1	O-methyltransferase
AFUA_4G14810	1.42	0.002	0.008	XP_751352.2	cytochrome P450 monooxygenase
AFUA_4G01550	1.41	0.033	0.000	XP_746302.1	aldehyde dehydrogenase
AFUA_3G01370	1.39	0.014	0.011	XP_748458.1	MFS transporter
AFUA_2G15450	1.38	0.017	0.030	XP_755880.2	conserved hypothetical protein
AFUA_1G11010	1.33	0.004	0.023	XP_752466.1	short chain oxidoreductase/dehydrogenase
AFUA_3G13700	1.32	0.006	0.016	XP_754254.1	transferase family protein
AFUA_5G01360	1.31	0.005	0.007	XP_748206.1	cytochrome P450
AFUA_6G05160	1.29	0.003	0.000	XP_747561.1	C2H2 transcription factor (Azf1)
AFUA_4G04170	1.25	0.021	0.009	XP_746603.1	conserved hypothetical protein
AFUA_4G11602	1.25	0.004	0.030	XP_001481570.1	conserved hypothetical protein
AFUA_4G14360	1.23	0.003	0.000	XP_751396.1	capsule associated protein
AFUA_6G06950	1.22	0.014	0.004	XP_750588.2	conserved hypothetical protein
AFUA_1G17240	1.21	0.030	0.044	XP_753092.1	C6 transcription factor
AFUA_5G08270	1.20	0.002	0.000	XP_753809.1	HAD superfamily hydrolase

AFUA_1G10420	1.20	0.011	0.009	XP_752408.1	hypothetical protein AFUA_1G10420
AFUA_1G14110	1.20	0.014	0.011	XP_752781.1	RNA polymerase subunit
AFUA_8G00150	1.20	0.028	0.009	XP_747189.1	conserved hypothetical protein
AFUA_1G17260	1.19	0.008	0.025	XP_753094.1	hypothetical protein AFUA_1G17260
AFUA_5G10890	1.19	0.011	0.000	XP_753557.2	DNA replication licensing factor Mcm6
AFUA_2G14790	1.18	0.017	0.004	XP_755811.1	conserved hypothetical protein
AFUA_6G07855	1.18	0.030	0.000	XP_001481458.1	conserved threonine rich protein
AFUA_5G10240	1.16	0.009	0.023	XP_753617.1	conserved hypothetical protein
AFUA_7G01030	1.16	0.036	0.000	XP_746828.1	calcium transporting ATPase (Pmc1)
AFUA_8G05280	1.15	0.004	0.000	XP_747330.1	RNA interference and gene silencing protein (Qde2)
AFUA_4G03690	1.14	0.043	0.041	XP_746556.2	aldehyde dehydrogenase family protein
AFUA_6G11300	1.13	0.013	0.003	XP_751014.1	integral membrane channel protein
AFUA_4G08600	1.12	0.005	0.000	XP_751967.1	aldehyde dehydrogenase
AFUA_3G07120	1.12	0.009	0.012	XP_754894.1	MFS transporter
AFUA_3G07945	1.10	0.018	0.042	XP_001481611.1	conserved hypothetical protein
AFUA_2G05380	1.09	0.023	0.030	XP_749668.1	C6 transcription factor
AFUA_1G16240	1.06	0.024	0.000	XP_752990.1	TIM barrel metal-dependent hydrolase
AFUA_5G13970	1.06	0.020	0.001	XP_753259.2	WD domain, G-beta repeat protein
AFUA_7G08650	1.04	0.021	0.019	XP_748750.1	conserved hypothetical protein
AFUA_1G17280	1.04	0.021	0.038	XP_753096.1	conserved hypothetical protein
AFUA_8G06810	1.02	0.022	0.000	XP_747480.1	hypothetical protein AFUA_8G06810
AFUA_2G16830	1.02	0.028	0.000	XP_756020.1	endonuclease/exonuclease/phosphatase family protein
AFUA_5G05590	1.01	0.022	0.000	XP_754072.1	aspartokinase

AFUA_4G12070	1.01	0.021	0.019	XP_751624.1	endosomal peripheral membrane protein (Mon2)
AFUA_3G08500	1.00	0.021	0.001	XP_754764.1	morphogenesis protein (Msb1)
