# Supplementary material for: Transcriptomic and Functional Studies of the RGS Protein Rax1 in Aspergillus fumigatus
Source: Pathogens. 2019 Dec 31;9(1):36. doi: 10.3390/pathogens9010036 (PMC7168642; doi:10.3390/pathogens9010036)
Supplement: Supplementary file 1 [file pathogens-09-00036-s001.zip › New folder/Table S2.pdf]

Table S2. Down-regulated genes in Δ*rax1* relative to WT (> 2.0-fold, $p < 0.05$).

Gene name	Log_2FC	*p*-value	*q*-value	Protein product	Protein name
AFUA_6G09680	-10.83	0.000	0.000	XP_750857.2	O-methyltransferase GliM
AFUA_7G04850	-6.67	0.000	0.022	XP_749025.1	aldo-keto reductase
AFUA_3G03570	-6.02	0.000	0.000	XP_748677.1	67 kDa myosin-cross-reactive antigen family protein
AFUA_7G05080	-5.63	0.000	0.001	XP_749002.1	C6 transcription factor
AFUA_7G06270	-5.28	0.000	0.000	XP_748883.1	cyanamide hydratase
AFUA_3G10850	-4.92	0.000	0.011	XP_754534.1	DUF821 domain protein
AFUA_7G05560	-4.71	0.000	0.029	XP_748953.1	hypothetical protein AFUA_7G05560
AFUA_3G00950	-4.27	0.000	0.000	XP_748416.1	ankyrin repeat protein
AFUA_3G03290	-4.22	0.000	0.000	XP_748648.1	conserved hypothetical protein
AFUA_3G03560	-3.92	0.000	0.000	XP_748676.1	pyridine nucleotide-disulfide oxidoreductase
AFUA_7G05190	-3.90	0.007	0.000	XP_748990.1	MFS alpha-glucoside transporter
AFUA_3G13120	-3.29	0.000	0.000	XP_754310.1	porphobilinogen deaminase Hem3
AFUA_2G14547	-3.28	0.000	0.004	XP_001481679.1	conserved hypothetical protein
AFUA_3G00370	-3.19	0.000	0.000	XP_748359.1	phosphoketolase
AFUA_8G01530	-3.09	0.000	0.020	XP_747053.1	HHE domain protein
AFUA_3G07410	-3.05	0.000	0.000	XP_754868.1	isoamyl alcohol oxidase
AFUA_4G00440	-2.97	0.000	0.020	XP_746412.1	short chain dehydrogenase
AFUA_7G05160	-2.96	0.002	0.000	XP_748993.2	fumarylacetoacetate hydrolase family protein
AFUA_7G04630	-2.94	0.000	0.000	XP_749047.1	hypothetical protein AFUA_7G04630
AFUA_3G14140	-2.90	0.000	0.025	XP_754211.1	metacaspase CasB
AFUA_7G01050	-2.82	0.004	0.038	XP_746826.1	salicylate hydroxylase

AFUA_7G05490	-2.80	0.000	0.004	XP_748960.1	conserved hypothetical protein
AFUA_2G17630	-2.77	0.001	0.034	XP_756098.1	conserved hypothetical protein
AFUA_3G01960	-2.74	0.000	0.002	XP_748516.1	conserved hypothetical protein
AFUA_3G01900	-2.68	0.000	0.000	XP_748510.2	conserved hypothetical protein
AFUA_5G09970	-2.63	0.000	0.035	XP_753643.2	67 kDa myosin-cross-reactive antigen family protein
AFUA_3G13620	-2.60	0.000	0.000	XP_754262.2	cupin domain protein
AFUA_7G05015	-2.58	0.000	0.045	XP_001481428.1	glyoxalase family protein
AFUA_3G11070	-2.48	0.000	0.041	XP_754512.1	pyruvate decarboxylase PdcA
AFUA_3G03220	-2.46	0.000	0.000	XP_748641.1	conserved hypothetical protein
AFUA_7G06940	-2.45	0.010	0.030	XP_748816.1	fungal specific transcription factor
AFUA_8G06590	-2.37	0.001	0.005	XP_747460.1	hypothetical protein AFUA_8G06590
AFUA_8G02280	-2.32	0.001	0.009	XP_746978.1	C6 transcription factor
AFUA_5G15060	-2.09	0.000	0.000	XP_753151.1	terpene synthase family protein
AFUA_3G00540	-2.05	0.000	0.001	XP_748374.1	MFS peptide transporter
AFUA_5G10020	-1.96	0.000	0.000	XP_753639.1	sensor histidine kinase/response regulator
AFUA_8G01820	-1.94	0.018	0.007	XP_001481397.1	hypothetical protein AFUA_8G01820
AFUA_5G15000	-1.94	0.000	0.000	XP_753157.1	arsenate reductase ArsC
AFUA_3G01590	-1.93	0.000	0.001	XP_748480.1	conserved hypothetical protein
AFUA_4G03280	-1.92	0.022	0.023	XP_746514.1	short-chain dehydrogenase/reductase family protein
AFUA_4G13860	-1.92	0.000	0.000	XP_751446.2	extracellular salicylate hydroxylase/monooxygenase
AFUA_7G01000	-1.91	0.013	0.005	XP_746831.1	aldehyde dehydrogenase
AFUA_8G01870	-1.90	0.048	0.000	XP_747019.1	hypothetical protein AFUA_8G01870
AFUA_3G02570	-1.87	0.000	0.000	XP_748578.1	polyketide synthase

AFUA_4G01322	-1.85	0.001	0.000	XP_746325.2	C6 transcription factor
AFUA_8G04510	-1.84	0.000	0.000	XP_747258.1	pirin
AFUA_3G11840	-1.83	0.000	0.000	XP_754437.1	NAD binding Rossmann fold oxidoreductase
AFUA_8G06280	-1.82	0.040	0.000	XP_747429.1	casein kinase family protein
AFUA_6G02360	-1.81	0.009	0.004	XP_747841.1	conserved hypothetical protein
AFUA_4G00910	-1.77	0.013	0.000	XP_746365.2	short chain oxidoreductase (CsgA)
AFUA_1G17020	-1.77	0.000	0.014	XP_753070.1	UDP-glucose dehydrogenase
AFUA_5G00300	-1.77	0.002	0.000	XP_748310.1	zinc-binding oxidoreductase
AFUA_8G02100	-1.76	0.001	0.021	XP_746996.1	beta-glucosidase
AFUA_6G02400	-1.74	0.002	0.018	XP_747837.1	MFS drug efflux pump
AFUA_2G01740	-1.72	0.000	0.005	XP_749309.1	sulfate transporter
AFUA_4G00840	-1.69	0.023	0.002	XP_746372.1	hypothetical protein AFUA_4G00840
AFUA_6G14520	-1.68	0.012	0.016	XP_751326.1	PTR family peptide transporter
AFUA_1G16350	-1.68	0.000	0.007	XP_753001.1	hypothetical protein AFUA_1G16350
AFUA_1G17040	-1.64	0.000	0.040	XP_753072.1	D-lactate dehydrogenase
AFUA_2G04190	-1.62	0.000	0.041	XP_749551.1	conserved hypothetical protein
AFUA_4G04318	-1.61	0.000	0.027	XP_001481547.1	copper resistance protein Crd2
AFUA_6G12120	-1.56	0.044	0.003	XP_751091.1	BNR/Asp-box repeat domain protein
AFUA_8G05600	-1.53	0.002	0.000	XP_747361.1	conserved hypothetical protein
AFUA_4G08970	-1.52	0.000	0.007	XP_751928.2	PAP2 domain protein
AFUA_3G14710	-1.50	0.009	0.003	XP_754154.1	toxin biosynthesis proten (Fum3)
AFUA_1G13110	-1.46	0.000	0.045	XP_752681.1	4-coumarate-CoA ligase
AFUA_3G02060	-1.45	0.000	0.012	XP_748525.2	MFS multidrug transporter

AFUA_7G04680	-1.43	0.015	0.000	XP_749042.1	conserved hypothetical protein
AFUA_2G12500	-1.41	0.000	0.003	XP_755582.1	MFS multidrug transporter
AFUA_3G06600	-1.38	0.000	0.022	XP_754946.1	siroheme synthase
AFUA_3G08450	-1.38	0.000	0.000	XP_754769.1	hypothetical protein AFUA_3G08450
AFUA_2G17360	-1.37	0.005	0.016	XP_756071.1	MFS monocarboxylate transporter (Mct)
AFUA_3G01670	-1.29	0.005	0.000	XP_748488.1	MFS hexose transporter
AFUA_3G10530	-1.23	0.000	0.000	XP_754566.1	protein serine/threonine kinase (Ran1)
AFUA_6G00310	-1.19	0.011	0.003	XP_731524.1	serine carboxypeptidase (CpdS)
AFUA_4G04670	-1.19	0.001	0.000	XP_746653.1	calcium permease family membrane transporter
AFUA_2G00880	-1.19	0.005	0.000	XP_749225.2	C6 finger domain protein
AFUA_2G04860	-1.18	0.002	0.012	XP_749617.1	DNA repair protein Rad4
AFUA_6G05350	-1.17	0.002	0.021	XP_747542.1	aspartic-type endopeptidase (OpsB)
AFUA_5G12580	-1.17	0.002	0.000	XP_753395.1	GTP binding protein (GTPBP1)
AFUA_6G12550	-1.16	0.002	0.045	XP_751133.1	mitochondrial carrier protein
AFUA_3G00330	-1.13	0.008	0.007	XP_748355.2	NAD dependent epimerase/dehydratase family protein
AFUA_7G07010	-1.11	0.020	0.000	XP_748810.1	hypothetical protein AFUA_7G07010
AFUA_1G11390	-1.10	0.002	0.045	XP_752504.1	cytochrome P450
AFUA_3G03190	-1.09	0.047	0.000	XP_748638.1	MFS multidrug transporter
AFUA_3G04050	-1.08	0.007	0.000	XP_748725.1	conserved hypothetical protein
AFUA_1G11910	-1.07	0.007	0.024	XP_752557.1	conserved histidine-rich protein
AFUA_3G08550	-1.03	0.012	0.005	XP_754760.1	short-chain dehydrogenase/reductase family protein
AFUA_1G17140	-1.02	0.012	0.034	XP_753082.1	voltage-gated K+ channel beta subunit (KCNAB)
AFUA_5G11330	-1.02	0.005	0.023	XP_753516.1	conserved hypothetical protein

AFUA_5G12620	-1.00	0.007	0.009	XP_753391.1	CorA family metal ion transporter
