# Supplementary material for: Transcriptomic and Functional Studies of the RGS Protein Rax1 in Aspergillus fumigatus
Source: Pathogens. 2019 Dec 31;9(1):36. doi: 10.3390/pathogens9010036 (PMC7168642; doi:10.3390/pathogens9010036)
Supplement: Supplementary file 1 [file pathogens-09-00036-s001.zip › New folder/Table S3.pdf]

Table S3. Oligonucleotides used in this study.

Name	Sequence (5'→3')	Purpose
oligo346	CCATGTGTGTCGAGTCCTTC	5' *ef1α* for qRT-PCR normalization
oligo347	GAACGTACAGCAACAGTCTGG	3' *ef1α* for qRT-PCR normalization
oligo691	CGATCTGTACCCCAACGAGT	5' *gliM* for qRT-PCR
oligo692	TTCTGGAACTTTGCCAGCTT	3' *gliM* for qRT-PCR
oligo1112	ACTCAGGTCATGGTGGTCAG	5' *casA* for qRT-PCR
oligo1113	AAGTCGACCGGGTAGATGAC	3' *casA* for qRT-PCR
oligo1114	ACGACGTCATCTACCCTGTC	5' *casB* for qRT-PCR
oligo1115	GCGAGGTTCGGTTCTTTCAA	3' *casB* for qRT-PCR
